# Contemporary multidisciplinary treatment of pregnancy-associated breast cancer

**DOI:** 10.1186/2193-1801-2-297

**Published:** 2013-07-03

**Authors:** Jane L Meisel, Katherine E Economy, Katherina Zabicki Calvillo, Lydia Schapira, Nadine M Tung, Shari Gelber, Sandra Kereakoglow, Ann H Partridge, Erica L Mayer

**Affiliations:** Brigham and Women’s Hospital, Boston, MA USA; Massachusetts General Hospital, Boston, MA USA; Beth Israel Deaconess Medical Center, Boston, MA USA; Dana-Farber Cancer Institute, Boston, MA USA

**Keywords:** Pregnancy, Breast cancer, Chemotherapy, Fetal outcomes, Multidisciplinary care

## Abstract

Breast cancer diagnosed during pregnancy poses unique challenges. Application of standard treatment algorithms is limited by lack of level I evidence from randomized trials. This study describes contemporary multidisciplinary treatment of pregnancy-associated breast cancer (PABC) in an academic setting and explores early maternal and fetal outcomes. A search of the Dana-Farber/Harvard Cancer Center clinical databases was performed to identify PABC cases. Sociodemographic, disease, pregnancy, and treatment information, as well as data on short-term maternal and fetal outcomes, were collected through retrospective chart review. 74 patients were identified, the majority with early-stage breast cancer. Most (73.5%) underwent surgical resection during pregnancy, including 40% with sentinel lymph node biopsy and 32% with immediate reconstruction. A total of 36 patients received anthracycline-based chemotherapy during pregnancy; of those, almost 20% were on a dose-dense schedule and 8.3% also received paclitaxel. 68 patients delivered liveborn infants; over half were delivered preterm (< 37 weeks), most scheduled to allow further maternal cancer therapy. For the infants with available data, all had normal Apgar scores and over 90% had birth weight >10^th^ percentile. The rate of fetal malformations (4.4%) was not different than expected population rate. Within a multidisciplinary academic setting, PABC treatment followed contemporary algorithms without apparent increase in maternal or fetal adverse outcomes. A considerable number of preterm deliveries were observed, the majority planned to facilitate cancer therapy. Continued attention to maternal and fetal outcomes after PABC is required to determine the benefit of this delivery strategy.

## Introduction

It is estimated that cancer complicates as many as 1 in 1000 pregnancies, with breast cancer as the most common associated malignancy (Smith et al. 2001). Contemporary management of pregnancy-associated breast cancer (PABC) encourages continuation of pregnancy during treatment. Limitations in published data describing the safety of cancer therapy in pregnancy create challenges and dilemmas in clinical decision-making for providers caring for these patients. Prospective evaluation of the safety of antineoplastic therapy during pregnancy is methodologically difficult; therefore, most published data consist of case and cohort series (Mir et al. 2008; Cardonick et al. 2012a; Loibl et al. 2012). Given the apparent rising incidence of PABC, perhaps due to trends in delaying pregnancy (Andersson et al. 2009; Litton & Theriault 2010), a greater understanding of the pathologic features, treatment options, and maternal and fetal outcomes in PABC is critical.

PABC has been defined as breast cancer diagnosed during pregnancy and up to one year postpartum. Although much PABC research has considered this group as a whole, it is worth considering two distinct subsets – those diagnosed during pregnancy, and those diagnosed in the postpartum period (Borges & Schedin 2011). Physicians caring for a pregnant patient with active breast cancer must select treatment options which maximize benefits to the patient while minimizing risks to the developing fetus. Given the advances in contemporary management of breast cancer, including use of taxanes, “dose dense” schedules, and sentinel lymph node biopsy, it is imperative to understand whether or not these new treatments can be safely administered during pregnancy.

With these considerations, a cohort of breast cancer patients treated during pregnancy was identified at an academic comprehensive cancer center. This study examined the distribution of tumor subtypes, treatment decisions, and maternal and fetal outcomes within the cohort, and evaluated the contemporary management of PABC supported by a multidisciplinary team.

## Methods

A retrospective cohort of patients diagnosed with breast cancer during pregnancy was created through a search of the Dana Farber/Harvard Cancer Center (DF/HCC) clinical database. Eligibility criteria included: a history of pathologically confirmed breast cancer diagnosed during pregnancy, at least two follow-up visits for treatment documented within the DF/HCC system (including the Dana-Farber Cancer Institute, Massachusetts General Hospital, Brigham and Women’s Hospital, and Beth Israel Deaconess Medical Center) to identify continuous care, and age > 18 years. Medical oncologists, breast surgeons, and maternal-fetal medicine specialists within the DF/HCC institutions were also queried to identify eligible patients treated in their practices. Comprehensive review of the DF/HCC database was used to gather patient information as well as data on short-term maternal and fetal outcomes. Statistical analysis was exploratory and descriptive in nature. Institutional Review Board approval was obtained prior to initiation of this study.

## Results

### Patient and disease characteristics

Seventy-four patients with PABC were identified who met inclusion criteria. Detailed demographic and disease characteristics are presented in Table 1. Most patients (62%) received their diagnosis after 2005. The median age at diagnosis was 34 years (range 25–40), with 63 (85.1%) patients aged 30 or older at the time of diagnosis. The timing of presentation varied with 25 (33.8%) patients diagnosed in the first trimester, 30 (40.5%) in the second trimester, and 19 (25.7%) in the third trimester. Sixty-eight of the 74 patients (91.9%) carried their infants to term. One patient miscarried and five pregnancies were electively terminated during the first trimester, prior to initiating cancer therapy.

**Table 1:** **Patient and tumor characteristics**

Demographic (N = 74)	Number	Percent
**Age at diagnosis (years)**		
Less than 30	11	14.9
30-34	26	35.1
35-39	29	39.2
≥40	8	10.8
**Median age at diagnosis:34 (range 25–40)**		
**Gestational age at diagnosis (weeks)**		
0-12 (1^st^ trimester)	25	33.8
13-27 (2^nd^ trimester)	30	40.5
28-40 (3^rd^ trimester)	19	25.7
**Race**		
White	63	85.1
Black	4	5.4
Hispanic	3	4.1
Asian	4	5.4
**Year of diagnosis**		
1996-1999	9	12.2
2000-2004	19	25.7
2005-2009	31	41.9
2010-2012	15	20.3
**Outcome of pregnancy**		
Delivery of liveborn infant(s)	68	91.9
Miscarried in 1^st^ trimester	1	1.4
Terminated in 1^st^ trimester	5	6.8
**Disease stage**		
0	1	1.4
I	19	25.7
II	42	56.8
III	10	13.5
IV	2	2.7
**Tumor subtype**		
ER/PR Positive	46	62.2
HER2 Positive	25	33.8
Triple Negative	14	18.9
**BRCA status**		
Genetic testing performed	50	67.6
BRCA 1 or 2 positive (N = 50)	6	12.0
BRCA 1 and 2 negative (N = 50)	44	88.0
Genetic testing not performed	24	32.4

Abbreviations: *ER* estrogen receptor, *PR* progesterone receptor, *HER2* human epidermal growth factor receptor 2.

The majority of patients (83.8%) were diagnosed with early-stage disease; only two had metastatic disease at the time of presentation. All were tested to evaluate tumor hormone receptor and HER2 status; 46 (62.2%) were ER/PR positive, 25 (33.8%) were HER2 positive, and 14 (18.9%) had triple-negative breast cancer. A total of 50 patients (67.6%) had BRCA testing performed, and in this subset, 6 (12.0%) tested positive for either a BRCA 1 or 2 mutation.

### Imaging

Most patients (67, 90.5%) underwent imaging to stage their disease while pregnant. A total of 54 (73.0%) underwent breast ultrasound, 30 (44.8%) underwent mammography, and 14 (20.9%) underwent chest or abdominal x-ray. A minority (8, 11.9%) underwent MRI of the breast, abdomen and/or spinal cord, and of this subset, about half received gadolinium. No patients underwent computed tomography while pregnant.

### Local therapy

Fifty of 68 patients (73.5%) who carried their pregnancies to term underwent surgery during pregnancy (see Table 2). Of this group, 39 underwent either lumpectomy or mastectomy, and 11 underwent more than one operation, resulting in a total of 37 lumpectomies and 25 mastectomies performed during pregnancy in the overall cohort. A total of 11 (17.7%) surgeries were performed during the first trimester and 35 (56.5%) in the second trimester. Immediate reconstruction was performed after 8 (32%) of the mastectomies during pregnancy. In those having surgery, sentinel lymph node biopsy was performed in 20 patients (40.0%), with 12 procedures employing radiolabeled tracer and 7 using blue dye.

**Table 2:** **Surgical therapy for pregnancy associated breast cancer**

Surgical therapy (N = 68 patients delivered live born infants, 50 patients underwent 62 surgical procedures)	Number	Percent
**Surgery during pregnancy (entire cohort N = 68)**		
Yes	50	73.5
No	18	26.5
**Gestational age at time of operation (N = 62)**		
0-12 weeks (1^st^ trimester)	11	17.7
13-27 weeks (2^nd^ trimester)	35	56.5
28-40 weeks (3^rd^ trimester)	16	25.8
**Type of operation performed (N = 50)**		
Lumpectomy	37	74.0
Mastectomy	25	50.0
**Immediate reconstruction after mastectomy (N = 25)**		
Yes	8	32.0
No	17	68.0
**Sentinel node biopsy performed (N = 50)**		
Yes	20	40.0
Methylene Blue tracer	7	35.0
Radiolabeled tracer	12	60.0
Unknown	1	5.0

### Systemic therapy

Thirty-six of the 68 patients (52.9%) who carried pregnancies to term received chemotherapy (see Table 3); 29 (80.6%) started chemotherapy in the second trimester and 7 (19.4%) started chemotherapy in the third trimester. No chemotherapy was administered in the first trimester. Decisions regarding agent selection and schedule were left to the discretion of the treating physician after consultation with patients, although opinion from multidisciplinary tumor board was considered in many cases as well. All 36 patients received doxorubicin and cyclophosphamide (AC) and 26 (72.2%) completed 4 cycles during pregnancy; of those receiving AC, 29 (80.6%) were treated on an every-3-week basis, and 7 (19.4%) received “dose-dense” AC every two weeks. Of these 7 patients, 4 received pegfilgrastim and 2 received filgrastim during pregnancy. Three patients (8.3%) received 12 cycles of weekly paclitaxel during pregnancy following AC. No patients received trastuzumab or endocrine therapy during pregnancy.

**Table 3:** **Systemic therapy for pregnancy associated breast cancer**

Systemic therapy (N = 68 patients delivered live born infants, 36 received chemotherapy therapy)	Number	Percent
**Chemotherapy during pregnancy (N = 68)**		
Yes	36	52.9
No	32	47.1
**Gestational age at initiation of treatment (N = 36)**		
0-12 weeks (1^st^ trimester)	0	0.0
13-27 weeks (2^nd^ trimester)	29	80.6
28-40 weeks (3^rd^ trimester)	7	19.4
**Chemotherapy regimen (N = 36)**		
Every-3-week AC	29	80.6
4 cycles	21	72.4
3 cycles	2	6.9
2 cycles	5	17.2
1 cycle	1	3.4
Dose-dense AC	7	19.4
4 cycles	5	71.4
2 cycles	2	28.6
AC × 4 + weekly paclitaxel (12 weeks)	3	8.3
Dose-dense AC	2	66.6
Every-3-week AC	1	33.4
**Neoadjuvant chemotherapy (N = 36)**		
Yes	8	22.2
**Growth factor support (N = 36)**		
Yes	6	16.7
Pegfilgrastim	4	66.7
Filgrastim	2	33.3

Abbreviations: *AC* doxorubicin, cyclophosphamide.

### Maternal outcomes

Of the 68 term pregnancies, 1 was complicated by intrauterine growth restriction (< 10^th^ percentile), 1 by gestational hypertension, 3 by gestational diabetes, and 3 by cervical shortening (defined as cervix < 3 cm at 22 weeks gestation). Other complications of pregnancy, such as preeclampsia and placental abruption, were not seen. Complications of chemotherapy, including anemia, neutropenia, and febrile neutropenia, were rare, and not more common with dose-dense therapy. One patient required a blood transfusion for anemia. A total of 13 patients (36.1%) treated with chemotherapy developed treatment-related neutropenia; but most cases were mild, with absolute neutrophil counts (ANC) >1000. One patient developed febrile neutropenia and mycoplasma pneumonia requiring hospitalization.

There were very few additional treatment-related complications; specifically, no pulmonary emboli or prolonged wound infections were seen. One patient with Factor V Leiden had a superficial blood clot. One patient experienced intraoperative preterm contractions at 30 weeks’ gestation; she underwent successful tocolysis and was induced closer to term.

### Fetal outcomes

A total of 68 of 74 patients delivered liveborn infants (Table 4). Over half of deliveries (37, 54.4%) were at < 37 weeks and thus were considered preterm. However, the majority of preterm deliveries (26, 70.2%) were planned in order to facilitate maternal therapy, e.g. continuation of chemotherapy. The remaining preterm deliveries (11, 29.7% of all preterm, 16.2% of all deliveries) were due to complications including spontaneous preterm labor or preterm premature rupture of membranes. Detailed birth information was available for 50 infants; missing information was from patients who delivered at outside hospitals. The rate of birth weight <10^th^ percentile was 8.0%. All infants had Apgar scores ≥ 7 at five minutes.

**Table 4:** **Fetal outcomes after treatment for pregnancy associated breast cancer**

Fetal Outcomes (N = 68 patients delivered live born infants)	Frequency	Percent
**Method of delivery (N = 68)**		
Method of delivery known	62	91.2
Spontaneous onset of labor	8	12.9
Induction of labor	38	61.3
Caesarian section	21	33.9
Method of delivery unknown	6	8.8
**Gestational age at delivery (N = 68)**		
Gestational age at delivery known	66	97.1
<34 weeks	4	6.7
34-36.9 weeks	33	50.0
>37 weeks	29	43.9
Gestational age at delivery unknown	2	2.9
**Weight for gestational age (N = 68)**		
Weight for gestational age known	50	73.5
SGA (<10^th^ percentile)	4	8.0
AGA	45	90.0
LGA (>10^th^ percentile)	1	2.0
Weight for gestational age unknown	18	26.5
**Apgar scores (N = 68)**		
Apgar scores known	50	73.5
≤7 at 5 minutes	0	0.0
≥7 at 5 minutes	50	100.0
Unknown	18	26.5
**Complications of delivery (N = 68)**		
Spontaneous preterm delivery	5	7.3
Arrested preterm labor	2	2.9
PPROM	5	7.4
Chorioamnionitis	1	1.5
Cord prolapse	1	1.5
Uterine atony	1	1.5
**Fetal Abnormalities (N = 68)**		
Cleft palate	1	1.5
VSD, club foot, hypospadias	1	1.5
ASD	1	1.5

Abbreviations: *SGA* small for gestational age, *AGA* average/appropriate for gestational age, *LGA* large for gestational age, *PPROM* preterm premature rupture of membranes, *VSD* ventricular septal defect, *ASD* atrial septal defect.

Three patients gave birth to infants born with congenital anomalies. One infant was found to have a cleft palate; the mother had other risk factors including tobacco and opiate use, and there was no chemotherapy exposure during pregnancy (Shaw et al. 1996). One infant had an atrial septal defect noted prior to initiation of chemotherapy at 31 weeks, after completion of fetal cardiac development. One infant was born with multiple abnormalities (ventricular septal defect, club foot, and hypospadias); the VSD and club foot were noted prior to initiating breast cancer therapy at 20 weeks, and hypospadias was additionally seen at delivery.

## Discussion

In this cohort of patients with PABC, contemporary therapies for breast cancer including “dose-dense” scheduling, taxane chemotherapy, growth factor support, immediate breast reconstruction, and sentinel lymph node biopsy, were utilized without significant rates of adverse maternal or fetal events. There was a low rate of maternal complications, and the overall rate of fetal abnormalities, 4.4%, was consistent with expected population rates (Parker et al. 2010). This series is one of the most modern cohorts of PABC patients in the current literature, with the majority of patients receiving their diagnosis during or after the year 2005.

Consistent with prior reports, and likely due to the young age of the patients, this PABC cohort was enriched for ER-negative tumors as well as BRCA mutations (Middleton et al. 2003; Elledge et al. 1993). However, patients’ clinicopathologic details did not differ greatly from those noted in other studies of non-pregnant young women diagnosed with breast cancer (Collins et al. 2012).

Almost all patients in the cohort underwent imaging to stage their disease while pregnant. Notably, nearly half of patients underwent mammography and over 10% underwent MRI scanning, with no apparent adverse effect on the fetus. The safety and feasibility of mammography during pregnancy have been reported (Kopans 1998; Yang et al. 2006), and it is unclear why rates of mammography were not higher in this population. The findings from this study support the safety of mammography during pregnancy; and suggest that MRI may have a role in select cases (Taylor et al. 2011).

Reports suggest that mastectomy or lumpectomy can be safely pursued at any point during a pregnancy with minimal risk to the fetus (Rosenkranz & Lucci 2005; Amant et al. 2010). However, first trimester surgery is often deferred, out of concern for higher risks of fetal complications (Moran et al. 2007). In this cohort, nearly one in five patients underwent surgery in the first trimester without apparent complications, suggesting the safety of surgical intervention during this time. Additionally, both sentinel lymph node biopsy and immediate reconstruction were utilized without apparent complication. Overall, very few surgical complications were observed, consistent with previous observations (Dominici et al. 2010). These data support the safety of contemporary surgical treatment paradigms in PABC, as long as practitioners are aware of the unique physiologic changes associated with pregnancy (Hill & Pickinpaugh 2008).

Although chemotherapy administration during the first trimester has been associated with risk of fetal defects, the use of certain chemotherapeutics in the second and third trimesters is generally considered safe without increase in the rates of fetal abnormalities (Loibl et al. 2012; Amant et al. 2010; Cardonick & Iacobucci 2004; Ring et al. 2005). The chemotherapy data presented in this study support the safety of administering traditional as well as more contemporary treatment regimens in this population. Dose-dense scheduling has an important role in modern adjuvant breast cancer treatment, however is not typically offered to women during pregnancy due to concerns regarding potential harm to the fetal bone marrow. A prior study of 10 pregnant patients treated with dose-dense AC found no difference in maternal or neonatal outcomes when compared with every three week dosing (Cardonick et al. 2012b). In this study, in the patients who received AC, including the significant minority who received a “dose-dense” schedule, there were no apparent short-term adverse effects on offspring.

Most prior studies evaluating chemotherapy in PABC have examined AC or FAC, but few studies have examined the safety of taxanes, which play an integral role in adjuvant chemotherapy regimens. In this cohort, 8.3% of patients who received AC also received 12 cycles of weekly paclitaxel during pregnancy without evident undesirable effects. To date, the use of taxanes in PABC has been reported primarily as case reports (Potluri et al. 2006; Nieto et al. 2006). In a recent review of this literature, it was noted that of 42 infants born to 40 patients exposed to taxanes during pregnancy, only one had a malformation possibly related to taxane use (Mir et al. 2010). Further expansion of this data is desired to confirm the safety of taxane-containing chemotherapy regimens after the first trimester.

In non-pregnant women, growth factors may be required during adjuvant chemotherapy, particularly with “dose-dense” schedules. There are few reports in the literature of patients receiving growth factors during pregnancy (Dale et al. 2003). Consistent with the prior publications, 6 patients in this study received growth factor support without apparent adverse effect on the fetus.

Although neither tamoxifen nor trastuzumab has been rigorously studied in pregnant patients, these important systemic agents are traditionally avoided in pregnancy out of concern for teratogenic effects. Case reports have described multiple fetal complications after prenatal exposures to these agents (Gottschalk et al. 2011; Braems et al. 2011; Witzel et al. 2008). Trastuzumab has been associated with oligohydramnios, and a boxed warning for teratogenicity has been issued, contraindicating the use of trastuzumab in pregnancy. As a short-term delay in the initiation of endocrine therapy likely does not adversely affect survival outcomes, particularly in early stage breast cancer, it is feasible to consider delayed initiation of tamoxifen until completion of pregnancy. However, given the growth kinetics of HER2 positive breast cancer, a delay in HER2-directed therapy may be more clinically significant and introduce potential risk of compromise in the care of these patients. Fortunately, current adjuvant regimens for HER2 positive breast cancer can begin with anthracycline-based chemotherapy, facilitating postponement of trastuzumab therapy until after delivery. Analysis of unintentional pregnancies during adjuvant trastuzumab clinical trials is in process (Azim et al. 2012), and an ongoing prospective study (MotHER pregnancy registry) is collecting data on pregnancies complicated by trastuzumab exposure.

In general, increased rates of maternal complications of pregnancy were not observed in this cohort, possibly reflecting early inclusion of a maternal-fetal medicine specialist in the multidisciplinary team. A notable finding was the observation that over half of deliveries occurred at < 37 weeks, and were considered preterm. In contrast, the current expected rate of preterm delivery in the United States is 12.2% (Martin et al. 2011). With the exception of one series suggesting rates of preterm delivery comparable to general population norms (Cardonick et al. 2010), most other published studies have also reported an increased risk of preterm delivery in patients treated with chemotherapy for PABC (Smith et al. 2001; Loibl et al. 2012; Van Calsteren et al. 2010). In this series, the majority of preterm deliveries were planned to facilitate maternal therapy, e.g. to allow continuation of chemotherapy. Only 16.2% experienced unplanned preterm delivery, a figure more consistent with national averages for non-PABC patients.

The question of planned premature delivery deserves examination. Although preterm delivery in this cohort did not appear to result in increased harm to the fetus, recent obstetrical data has suggested even late preterm delivery (34–37 weeks) or near term delivery (37–39 weeks) can be associated with adverse outcomes in the fetus (Hibbard et al. 2010; Shapiro-Mendoza et al. 2008). In addition, a recent evaluation of children exposed to chemotherapy in utero has noted lower cognitive development for those born preterm than for those born full term (Amant et al. 2012). Given the apparent safety of chemotherapy during pregnancy, especially in the third trimester, the risks of late preterm delivery must be carefully considered when planning the timing of delivery. In these situations, a careful discussion is encouraged between the patient, the maternal-fetal medicine specialist, and the medical oncologist so that delivery timing minimizes risks to both mother and fetus.

Strengths of this study include utilization of a large academic comprehensive cancer center, and evaluation of a sizeable number of pregnant breast cancer patients treated with more contemporary treatment regimens than have been previously published. In addition, a systematic approach was used to assemble the cohort rather than solely relying on physician recall or patient self-enrollment, eliminating a source of bias. Finally, the majority of patients were treated for their breast cancer and delivered their infants at the same institution, allowing for data capture for peripartum as well as maternal outcomes.

A limitation of this study is the lack of follow-up data in exposed infants; data is limited to in utero and perinatal outcomes. Obtaining retrospective follow-up data is complicated by loss to follow-up and limitations of self-reported data. However, prospective long-term follow-up studies have generally demonstrated favorable outcomes in exposed offspring (Aviles & Neri 2001; Hahn et al. 2006). Another limitation of this study is sample size; while this cohort is large compared to other published cohorts of PABC, the sample size is small in absolute terms, making it difficult to generate definitive conclusions. In addition, the study was limited to women treated in a large academic tertiary care center, which introduces the possibility of selection bias. Data on socioeconomic status was not available, but it is possible that a higher degree of social and financial support may have impacted not only treatment choices but also patient outcomes.

This retrospective study of PABC demonstrates the feasibility of optimal treatment through multidisciplinary care team management. Continued prospective data collection and case series publication may improve comfort with using contemporary treatment paradigms to care for women in the difficult situation of having been diagnosed with breast cancer while pregnant. When making treatment decisions for these patients, attention to multidisciplinary team management is necessary to appropriately balance perinatal care of mother and infant. Further data collection and investigation will improve understanding of and treatment paradigms for breast cancer diagnosed during pregnancy.
